# Malaria and COVID-19: commonalities, intersections and implications for sustaining malaria control

**DOI:** 10.11604/pamj.supp.2020.37.1.25738

**Published:** 2020-09-01

**Authors:** Ikeoluwapo Oyeneye Ajayi, Olufemi Olamide Ajumobi, Catherine Falade

**Affiliations:** 1 Department of Epidemiology and Medical Statistics, College of Medicine, University of Ibadan, Ibadan, Nigeria,; 2 Epidemiology and Biostatistics Research Unit, Institute for Advanced Medical Research and Training, College of Medicine, University of Ibadan, Ibadan, Nigeria,; 3 School of Community Health Sciences, University of Nevada, Reno; Reno, USA,; 4 National Malaria Elimination Programme, Federal Ministry of Health, Abuja, Nigeria,; 5 Department of Pharmacology & Therapeutics, College of Medicine, University of Ibadan, Ibadan, Nigeria

**Keywords:** COVID-19, malaria, integrated control, community testing, surveillance, pandemic

## Abstract

The devastating impact of infectious disease outbreaks and pandemics on health systems could be overwhelming especially when there is an overlap in clinical presentations with other disease conditions. A case in point is the disruptive effect of the Ebola Virus Disease outbreak on health service delivery and its consequences for malaria management in the affected West and Central African countries between 2014 and 2016. This could be the case with the current infectious disease pandemic (COVID-19) the world is experiencing as malaria illness shares many symptoms with COVID-19 illness. Caused by a novel coronavirus, severe acute respiratory syndrome coronavirus 2 (SARS-CoV-2), COVID-19 is reported to have originated from Wuhan city, China in December 2019. COVID-19 was declared a Public Health Emergency of International Concern on 30 January 2020 and declared a pandemic on March 11, 2020 by the World Health Organization (WHO). Practically, all community infrastructure has been activated in affected countries in response to COVID-19. However, the deployment of huge resources in combating COVID-19 pandemic should not be a missed opportunity for the advancement of infectious diseases control including malaria. This calls for conscious and heightened effort to sustain the gains in malaria control. The WHO has emphasized that the response to the COVID-19 pandemic must utilize and strengthen existing infrastructure for addressing malaria and other infectious diseases globally. Leveraging these to maintain malaria control activities in endemic countries could boost and help to sustain the gains in malaria control in accordance with the 2016-2030 Global technical strategy for malaria (GTS) milestones. In addition, it will help to keep the “High burden to high impact” (HBHI) and other initiatives on track. This article highlights the commonalities of the two diseases, discusses implications and recommendations to support decision making strategies to keep malaria control on track in the COVID-19 pandemic era.

## Essay

**Epidemiology of COVID-19:** an outbreak caused by a novel coronavirus, severe acute respiratory syndrome coronavirus 2 (SARS-CoV-2) was announced in Wuhan, China, in early December 2009 and the disease was later referred to as COVID-19 [1]. The disease is transmitted through respiratory droplets and contact routes [2]. It is also considered airborne and there is some evidence that COVID-19 infection also infects the intestine and may be present in feces [2]. As the virus spreads across the globe, different countries are in different stages of fighting the outbreak. It has been reported in all continents, except for Antarctica, and the figure has been steadily rising around the world [2]. Globally, 23, 618,698 persons have been infected with over 800,000 deaths (813,114) and in the Africa continent, over 1,187,937 cases and 27,779 deaths have been reported as at 24 August 2020 (8:00:00a.m) [3] with an upward trend. Malaria-endemic countries in all WHO regions have reported cases of COVID-19. In the WHO African Region, which carries more than 90% of the global malaria burden, 47 countries had reported approximately 1,003,435 cases, and 20, 398 deaths of the disease as of 24 August 2020 (6:00:00a.m) [3]. COVID-19 is characterized by a highly contagious infection with the entire population at risk and a higher risk of severe symptoms experienced by elderly and those with underlying health conditions such as immunocompromised state, diabetes mellitus, cardiovascular diseases, hypertension, chronic lung and renal diseases, liver disease, cancer and severe obesity. The affectation of children is also on the increase accounting for 1 to 10 percent of diagnosed cases [4]. Recently a clinical state similar to Kawasaki disease has been linked to COVID-19 in children [5].

**Malaria epidemiology:** malaria is caused by parasites of the genus *Plasmodium* transmitted through the bite of infected female *Anopheles* mosquitoes. It is a major cause of illness and death especially in sub-Saharan Africa. In 2018, 228 million cases and 405 000 deaths occurred globally [6]. Of these, 93% of cases were in sub-Saharan Africa across 38 African countries, 11 million were pregnant women malaria cases; and consequently 900,000 children were born with a low birth weight due to malaria in pregnancy. Children under five accounted for nearly two-thirds of reported deaths worldwide [6]. Poorer, marginalized and more rural populations are at greater risk, in endemic countries [7]. The parasites go through life cycles in both human and mosquitoes before causing symptoms in the sufferer; or it may remain seemingly asymptomatic, causing anemia and stunted growth in children and adverse pregnancy outcomes in pregnant women. Malaria affects all ages in endemic settings as an acute uncomplicated illness. However, children, pregnant women and the non-immune being vulnerable; suffer from severe malaria commonly seen with *P. falciparum* infection, unlike in areas of seasonal or epidemic prone where all ages are affected [8]. Asymptomatic carriers of malaria are more prevalent in high endemic areas serving as reservoirs of gametocytes for sustaining malaria transmission through *Anopheles* mosquitoes [9].

**COVID-19 pandemic issues and implications for malaria morbidity and mortality:** there are some COVID-19 issues that have similarities with malaria infection or may impact on malaria morbidity and mortality due to disruption in the milieu in which it occurs. These include the clinical presentation, the control measures, transmission, treatment and stigma.

**Clinical presentation:** COVID-19 exhibits similarities in clinical presentation [1-3,8,10-16] and empirical treatment [10,17] with malaria. In addition, they share some pathophysiological characteristics which supports the overlap in clinical presentations [12,14] (Table 1). The similarity in high occurrence of COVID-19 and malaria in densely populated/urban slum and rural areas is attributed to poverty, poor infrastructure, poor access to health, limited health manpower and pre-existing co-morbidities [18]; which favor high transmission of both diseases even though through different modes. The similarities in clinical presentations of these two diseases constitute a danger both ways. Attention has shifted to COVID-19 with malaria cases not receiving the requisite attention and attendant risk of missed or delayed malaria diagnosis and treatment. Fever is a cardinal symptom of COVID-19 and malaria, caregivers are bound to get confused with choice/practice of self-medication for malaria with the advent and awareness of COVID-19. Most deaths from severe malaria occur within the first 24 to 48 hours of symptoms occurrence if not treated [10]. The overlap of COVID-19 symptoms and malaria may lead to delay in treatment as a result of the testing guideline which has similar symptoms to malaria listed. Referring a patient with symptoms of malaria for COVID-19 testing may cause delay and testing negative for COVID-19 with no concomitant result of malaria test may be frustrating and may preclude efficacious treatment or delay treatment. On the other hand, misdiagnosis of COVID-19 as malaria coupled with denial of histories of travel to affected states or countries by patients have led to inadvertent exposure and death of healthcare workers and patients [19]. The use of call-in toll-free number to mitigate physical visits to health facilities and prevent transmission of infection has not been effective [20]. This has grave implications for severe malaria patients and the pervading fear of contracting COVID-19 in health facilities is a concern. Thus, an integrated approach to management of febrile illnesses and adopting efficient telehealth services, is critical [21]. Another threat to the severity of both diseases is the high prevalence of malnutrition, anemia, malaria, HIV/AIDs, and tuberculosis in the population which reduces immunity and increase susceptibility to severe diseases even in the younger age group which may be different from the narrative in developed countries. Hence, malaria endemic countries should bear this in mind and plan to mitigate effectively [22].

**Table 1:** comparison of Clinical presentations and pathophysiology of severe disease in COVID-19 and Malaria

		COVID-19	Malaria	
**Symptoms**	**Possible**	Asymptomatic, Pre-symptomatic, mild to critical, rapid progression to severe disease	Asymptomatic, mild to critical, rapid progression to severe disease	
	**Commonly**	Flu-like symptoms fever, headache, chills, myalgia, vomiting; diarrhea and cough (commoner in children for malaria)		
	**Severe/Critical**	Trouble breathing		Dehydration from excessive vomiting, severe anemia, prostration, convulsion, coma (unconsciousness), hyperbilirubinemia in liver injury, pulmonary complications that can lead to Acute Respiratory Distress Syndrome (ARDS), hypoxia, cyanosis, multiorgan dysfunction and disseminated intravascular coagulation, death
		Persistent pain or pressure in the chest		
		ARDS		
		Hypoxia		
		Cyanosis		
		Multiorgan dysfunction		
		Disseminated Intravascular Coagulation		
		Death		
		ARDS occurs in 20% of people who gets COVID-19		Malaria-ARDS occurs in 25% of adults, 40% (children), 29% (pregnant women), 21-23 % (non-immune patients)
	**Others**	Loss of taste and smell		Taste impairment - Bitter or metallic taste
	**Outcomes**	Fatal in elderly, medical co-morbidities, mild in children		Fatal in children, pregnant women, immune-compromised
		More in densely populated / urban slums and in racial minorities (blacks, Hispanics)		More in rural and densely populated / urban slums
**Treatment**		Empirical treatment with hydroxychloroquine/chloroquine, Azithromycin and other adjuvants		Chloroquine in countries were Plasmodium is still sensitive and artemisinin combination therapy in countries where there is chloroquine resistance
**Incubation period**		Average 5-6 days, Range:2 to 14 days		Average 7-14 days, Range 7-30 days
**Pathophysiology of severe disease**		A cytokine storm triggers an exaggerated inflammatory response that may damage the liver, blood vessels, kidneys, and lungs, and increase formation of blood clots throughout the body. ARDS from pulmonary thrombosis consequent to cytokine / inflammatory storm		
		Thrombosis in other organs causing multi-organ dysfunction/failure and/or disseminated intravascular coagulation (DIC) as may occur in MA-ARD.		

**Community testing:** the stage 3 transmission phase in COVID-19 means everybody stands the risk of being infected with source not identified [23]. There are (unknown) infected persons within the community who could infect other people, most of whom are asymptomatic. These asymptomatic persons and those in pre-symptomatic phases of COVID-19 as well as asymptomatic persons with malaria are a major challenge in curbing the transmission of these infections. For COVID-19 pandemic, community transmission is evident from guidelines-based testing among people with symptoms such as fever, cough or those diagnosed with influenza like illnesses or severe acute respiratory illnesses (SARI). Persons without symptoms are also prioritized by health departments or clinicians, for any reason, including public health monitoring, sentinel surveillance, or asymptomatic individuals undergoing screening according to state and local plans [24]. Thus, community testing for COVID-19 has commenced in malaria endemic countries. Testing has been limited in less developed countries due to high cost of diagnosis and inadequate test kits thus, was limited to symptomatic individuals and their contacts initially [25]. The transition to COVID-19 community testing has brought about an increase in detection of new cases as expected at this phase. However, there is a missed opportunity to integrate testing for malaria, a strong differential. The current gold standard for the treatment of malaria is laboratory confirmation (microscopy or rapid diagnostic test- RDT) before administration of Artemisinin-based Combination Therapy (ACT) [11]. However, the need for presumptive diagnosis in the era of COVID-19 has been allowed by the WHO in the wake of the COVID-19 pandemic [26]. Integrated testing for suspected Malaria-COVID-19 co-infection will increase access to and motivation for malaria rapid testing and treatment, enhanced surveillance and also reduce the anxiety experienced by people with symptoms but could be COVID-19 negative or COVID-19 malaria co-morbid patients. The WHO reiterated that response to the COVID-19 pandemic must utilize and strengthen the existing infrastructure for addressing malaria and other infectious diseases globally [26].

**Treatment of COVID-19 and the implications for malaria treatment:** the treatment of COVID-19 has been a challenge and there is currently no definitive curative medication. Current medical management is largely supportive with no targeted therapy available. Empirical treatment with repurposed drugs such as lopinavir-ritonavir, remdesivir, chloroquine (CQ), hydroxychloroquine, and azithromycin is ongoing [20]. Hydroxychloroquine (HCQ), a less toxic metabolite of chloroquine, an antimalarial; is used to treat immune-mediated diseases such as systemic lupus erythematosus, rheumatoid arthritis, juvenile idiopathic arthritis and Sjogren's syndrome [17]. There are mixed results on the effectiveness of CQ/HCQ. Although antiviral activity of CQ and HCQ against SARS-CoV-2 has been reported [27], there is insufficient evidence in-vivo, to recommend for use outside of clinical trials in the current pandemic. The specific drugs used by countries as empirical treatment of this disease have not been documented officially in many instances to know the extent of CQ/HCQ use. There are ongoing clinical trials, including the WHO solidarity trial, to find effective treatment for COVID-19. The use of CQ/HCQ in malaria endemic region for COVID-19 treatment has implications for established CQ resistance (pfCRT), tolerance based on immunological memory of past use and what evidence exists for its pre-exposure prophylaxis [19]. The use of CQ for COVID-19 might derail the enforcement of the ban of its use as an antimalarial with resultant irrational prophylactic use, chloroquine toxicity and deaths [28]. Home management of malaria (HMM) or Community Case management of Malaria and integrated community case management (iCCM) are current initiatives for increasing access to antimalarial treatment in underserved areas. The implementers of these are likely to continue to attend to febrile illnesses among children despite the movement restriction necessitated by control measure for COVID-19. There is a high risk of transmission of COVID-19 between these implementers and their patients especially now that the level of awareness of COVID-19 is still sub-optimal among rural dwellers. Movement restriction impacts the supply chain for malaria commodities with resultant use of alternative medicines which threatens the gains in malaria control in the community and near homes.

**COVID-19 preventive measures and implications for malaria control:** restriction of movement, cessation of interstate movement, intermittent health services, reduced visits to health facilities due to fear of getting infected with and being diagnosed of COVID-19, stigmatization of COVID-19 treatment centers, may culminate in ineffective treatment of malaria, other diseases and health conditions. These discourage pursuance of health care and may impact malaria morbidity and mortality [21]. The current lockdown impacts environmental management, encourages mosquito breeding which is made worse with the arrival/onset of the rainy season. This means that malaria cases will rise rapidly and peak malaria cases in 2020 may coincide with the ongoing COVID-19 pandemic. Efforts to ameliorate poverty during this pandemic have been fraught with the absence of reliable databases of individual and family incomes, as well as absence of traceable home and business addresses in some low-to-middle income countries such as Nigeria, which makes it difficult to effectively deploy government palliatives to those who really need them [29]. Thus, the poverty-malaria cycle may be aggravated. Heightening this is the fact that within the population is the high prevalence of malnutrition, anemia, malaria, HIV/AIDs, and tuberculosis.

Additionally, the supply chain for malaria commodities, insecticide-treated net (ITN) campaigns and indoor residual spraying have been impacted by the lockdown [26]. The pervading palpable fear of inadequate malaria commodities is notable considering the forecasted doubling of malaria deaths in sub-Saharan Africa in 2020 and thus, WHO/RBM issued a technical guidance on maintaining malaria services in COVID-19 settings [26]. The republic of Benin, the Democratic Republic of the Congo, Sierra Leone and Chad initiated ITN campaigns during the pandemic and other countries can learn lessons to do same as safely as possible [3,26]. The restrictive measures for COVID-19 has implications for malaria surveillance - The reduced healthcare visits, access to care and case detection limit reporting [6]. Thus, the concern on how to get reliable malaria surveillance data during COVID-19 pandemic. This calls for novel strategy or adapting current system to fit the COVID-19 surveillance system in view of integration.

Protection of health workers and impact on health care system: frontline and healthcare workers are at greater risk and must be assured of adequate protection to continue delivering vital health services. There has been inadequate supply of protective wears (personal protective equipment-PPE) for health workers in many affected malaria endemic countries [30]. This coupled with social distancing and fear may limit the motivation of health workers to provide malaria diagnosis and treatment services [21]. In addition to personnel, there is also a limitation to effective management of diseases in general at health facilities, and particularly at this time, COVID-19 and malaria. The health system in the African context is unique. There are population structure differences, high prevalence of endemic diseases and the double burden of disease, with health systems that are stretched thin with minimal critical care capacity [22]. The fear of this impact is already evident following alarm from experts in other diseases on the possible ongoing increase in mortality from non-COVID-19 diseases including malaria during this pandemic partially due to healthcare workers’ refusal to administer treatment and poor access to health facility and health professionals [11]

## Conclusion

The COVID-19 pandemic remains a global nightmare. Inappropriate malaria treatment remains a possibility in the current community transmission phase of the pandemic. Despite restrictive measures for COVID-19 control, there are immense opportunities as well for malaria control. Leveraging existing system and resources is very important at this time and will support both the control of malaria and COVID-19 especially in rural and hard-to-reach areas. These include integrated testing, surveillance, ITN distribution, and malaria prevention and health promotion messaging leveraging focused systemic efforts for COVID-19. While we anticipate increase in the availability of rapid test for COVID-19, an integrative approach can help mitigate the rising burden of malaria and avert the reversal of gains in malaria control. Malaria endemic countries simply cannot afford a lapse in Malaria control at this time. It is imperative that the WHO and End Malaria Partnership “High burden to high impact” (HBHI) initiative to reignite the pace of progress in the global malaria fight is not derailed as a consequence of the COVID-19 pandemic on the existing health infrastructure and service delivery.

### Recommendations

*Prioritizing reduction of malaria transmission is vital:* to avert upsurge of malaria occurrence at this peculiar period, vector control needs to be encouraged. The use of ITNs should be emphasized via mainstream and social media. Children should be kept indoors by evening time to avert mosquito bites. Mosquito breeding sites should be eliminated. Those with bushy environment should employ gardeners but appropriate PPE should be provided and used. Integrated community testing for COVID-19 and malaria should be adopted for early diagnosis of infection and appropriate management. One question arising from this is “should asymptomatic malaria be treated” in the course of community diagnosis? A robust COVID-19 response for the continent will need to take these factors into account and include community engagement, health leadership, and involvement of youth and religious leaders to drive containment. At the health system level, temporary repurposing and reorganizing of the health care system will be key to increasing critical care capacity during the response, focusing on what we have as we move forward.

*Leveraging existing systems for Integration of COVID-19 and malaria control:* door-to-door distribution of ITN and/or integration with economic palliatives distribution where feasible while distributors are provided appropriate protective measures for COVID-19 should be explored. Existing infrastructure for COVID-19 supplies distribution should be leveraged for malaria commodities distribution especially in rural and hard-to-reach areas. The proactive surveillance for COVID-19 should be used to strengthen malaria and other diseases surveillance in order to address global health security.

**Community sensitization on fever management in the wake of malaria season:** awareness and health education on symptoms of both diseases and early fever diagnosis, prompt presentation at health facility and efficacious treatment could be strengthened using mainstream and social media. The likely surge of febrile illness during the rains and the accompanied fear of COVID-19 should be addressed. The populace should be reminded that the drug of choice for uncomplicated malaria is ACT. A robust malaria response should include community engagement, health leadership, and involvement of youth and religious leaders just as it is been done to drive COVID-19 containment. Thus, integrated messaging, risk communication and prevention education is desirable. However, this should be guided to avoid confusion in the messages which could result in messages for one disease pulling down the other.

**Protecting health workers and increasing access to health care:** face masks for clinic attendees and personal protective equipment for health workers should be made available to allay the pervading fear associated with health service access and delivery especially for children and pregnant women who are prone to malaria mortality. Health care service providers should adhere strictly to diagnosis and treatment guidelines in managing patients presenting with COVID-19-like symptoms, in order to lessen their risks of infection and mortality from non-COVID-19 febrile illnesses including malaria in patients. In addition, the private sector and community healthcare providers including patent and proprietary medicine vendors should be re-trained on management of febrile illnesses, use of PPE in the face of COVID-19 and provided job aids such as algorithm for screening. Furthermore, malaria diagnosis and treatment commodities should be readily available.

**Future research:** many countries are learning more about COVID-19 as the epidemic is progressing. Research is needed to outline the epidemiology and course of the infection in individual countries in order to effectively provide a focused management plan for the disease and other non-COVID-19 diseases including malaria. In addition to the ongoing clinical trials on drugs and herbal preparations for COVID-19, the following research topic areas/questions are suggested to be researched in relation to malaria: i) evaluation of the efficacy of chloroquine/HCQ in the treatment of COVID 19 alone or in the treatment of malaria in Malaria-COVID-19 co-infected patient? ii) What is the tolerance and efficacy of ACT used in combination with CQ or HCQ in malaria-COVID-19 co-infection? iii) Can other effective antimalarials such as dihydroartemisinin-piperaquine be used for treatment of COVID-19 malaria co-infection? iv) Can the current surveillance for COVID-19 be an opportunity to strengthen malaria surveillance? v) What is the prevalence of malaria-COVID-19 co-infection? vi) What is the effect of malaria on the course/prognosis of COVID-19? vii) What is the severity of malaria when it co-exists with COVID-19? viii) What is the mortality rate of prevalent non-COVID-19 diseases during the pandemic?
